# Factors that influence adherence to surgical antimicrobial prophylaxis (SAP) guidelines: a systematic review

**DOI:** 10.1186/s13643-021-01577-w

**Published:** 2021-01-16

**Authors:** Sarah Hassan, Vincent Chan, Julie Stevens, Ieva Stupans

**Affiliations:** 1 Pharmacy, School of Health and Biomedical Sciences, RMIT University, Bundoora, Victoria Australia; 2 School of Pharmacy and Medical Sciences, University of South Australia, Adelaide, South Australia Australia; 3 Adelaide Medical School, Faculty of Health and Medical Sciences, University of Adelaide, Adelaide, South Australia Australia

**Keywords:** Surgical antimicrobial prophylaxis, Guideline adherence, Personal barriers, Organisational barriers, Multifaceted interventions

## Abstract

**Background:**

Despite the extensive research that has been conducted to date, practice often differs from established guidelines and will vary between individuals and organisations. It has been noted that the global uptake of local and international surgical antimicrobial prophylaxis (SAP) guidelines is poor with limited research investigating factors that affect guideline adherence. The purpose of this systematic review was to determine the reported barriers and enablers to the adherence of SAP guidelines.

**Methods:**

A search of the literature was performed using four electronic databases (CINAHL, EMBASE, PubMed and SCOPUS) for articles published in the English language from January 1998 to December 2018. Articles were included if they were solely related to SAP and discussed the barriers or enablers to SAP guideline adherence. Articles that assessed the adherence to a range of infection control measures or discussed adherence to antibiotic treatment guidelines rather than SAP guidelines were excluded from this review. Barriers and enablers were mapped to the Theoretical Domains Framework (TDF). The Mixed Methods Appraisal Tool was used to assess the quality of included studies.

**Results:**

A total of 1489 papers were originally retrieved, with 48 papers meeting the eligibility criteria. Barriers and enablers were mapped to 11 out of 14 TDF domains: knowledge, skills, social/professional role and identity, beliefs about capabilities, beliefs about consequences, reinforcement, memory, attention and decision processes, environmental context and resources, social influences, emotion and behavioural regulation. Barriers were further categorised into personal or organisational barriers, while enablers were arranged under commonly trialled interventions.

**Conclusions:**

There are numerous factors that can determine the uptake of SAP guidelines. An identification and understanding of these factors at a local level is required to develop tailored interventions to enhance guideline adherence. Interventions, when used in combination, can be considered as a means of improving guideline use.

**Supplementary Information:**

The online version contains supplementary material available at 10.1186/s13643-021-01577-w.

## Background

Surgical site infections (SSIs)—infections that occur at or near the site of a surgical incision within 30 days of procedure or within 90 days of prosthesis implantation—are classified as one of the most common types of nosocomial infections [1, 2], accounting for up to 38% of infections in surgical patients [3]. SSIs are often associated with a greater length of hospital stay, hospital readmissions, increased health care costs and mortality [4–6]. Surgical antimicrobial prophylaxis (SAP), the administration of antibiotics immediately prior to surgery, is a key strategy used to help prevent the development of post-operative infections, namely SSIs [7]. Whilst infection control practices such as operating room ventilation, surgical instrument sterilisation and ensuring adequate skin preparation may also play a role in preventing SSIs [3, 8, 9], the use of SAP has been pivotal in decreasing infection rates [10, 11].

Studies have focused on establishing the criteria that determine the appropriateness of SAP, with recommendations being updated by key bodies such as the Centers for Disease Control and Prevention (CDC) and the World Health Organization (WHO) [8, 9, 12]. In Australia, further guidance is provided by the Australian Commission on Safety and Quality in Health Care via the Antimicrobial Stewardship Clinical Care Standards. This standard provides statements on the delivery of care to a patient with bacterial infections as well as how antibiotics should be prescribed for SAP [13].

SAP recommendations are often presented in the form of clinical practice guidelines, with suggestions on the appropriate prescribing and administration of antibiotics. Optimal SAP is dependent on fulfilment of the following key quality indicators: correct selection of antimicrobial for indication, administration of correct dose via correct route, administration of preoperative antibiotics at the correct time with intraoperative doses given at the correct interval and administration of SAP for the recommended duration [7, 14].

Clinical practice guidelines—statements that assist practitioners in their decision making and reflect the most current evidence-based research—are recommended to be used alongside a clinician’s judgement in determining the best course of action for patients [15]. However, despite the evidence that is presented in such documents and the established benefits of using SAP, multiple audits have indicated that adherence rates to SAP recommendations is often suboptimal [16–19]. Studies have underlined non-concordance to many of the quality indicators, particularly a lack of adherence to timing of administration [16, 20, 21]. Extended duration of prophylaxis has also been documented and is of significant concern as it can contribute to growing antimicrobial resistance [16–18, 20]. Poor adherence to guidelines, arising from evidence-practice gaps, can lead to suboptimal health care, increased patient harm, diminished quality of life and unnecessary costs [22].

Current recommendations listed in CDC and WHO guidelines for the prevention of SSIs advocate for no further doses to be administered once incision has been closed for most procedures, while suggesting a limited duration of use to 24 h for procedures where evidence is lacking [8, 9, 12]. In addition to guidance provided for appropriate SAP, the CDC and WHO guidelines also list pre-, intra- and postoperative measures for the prevention of SSIs. Key measures associated with SSI prophylaxis preoperatively include surgical hand preparation, preoperative bathing with plain or antimicrobial soap and surgical site preparation with chlorhexidine gluconate-based antiseptic solutions [8, 12]. Maintaining normal body temperature and optimal perioperative blood glucose concentrations is recommended intraoperatively, with the use of standard wound dressings recommended over advanced dressings in the postoperative setting [9, 12].

Knowledge translation is required to ensure that science research is transferred to the clinical setting. This has been recognised as a complex and slow process, with estimates of a 17-year time lag between research and practice [23, 24]. In addition, the translation of quality evidence-based research has been identified as challenging for health care professionals [22]. The uptake of guidelines is inconsistent across various settings [25], with its utilisation also considered slow and unpredictable [26–28]. It has been reported that guidelines are followed in 67% of decisions, although this is highly variable between physicians and guidelines [27].

Successful implementation of practice change interventions requires an understanding of the personal and organisational factors that influence behaviour. The Theoretical Domains Framework (TDF) has previously been used in health care settings to explore the determinants of guideline use [29, 30]. TDF contains 14 domains which highlight how the interplay of individual, social and environmental factors may influence behaviour [31]. By understanding these factors through the lens of TDF, tailored interventions can be designed to address these factors and thus promote behaviour change.

Despite the knowledge of barriers and enablers to clinical guideline use in general, little is known regarding the determinants of SAP guideline uptake. Thus, the aim of this review was to identify the barriers and enablers to adherence of SAP guidelines in order to provide health care providers with a theoretically derived understanding of how to improve adherence to guidelines. The findings of this review may help improve the understanding of the personal and system-based factors that hinder the uptake of SAP guidelines, whilst also highlighting trialled interventions that can be employed by organisations in order to increase guideline uptake.

## Methods

### Search strategy

Using the Preferred Reporting Items for Systematic Reviews and Meta-Analyses (PRISMA) guidelines (Additional file 1) [32], a detailed literature search was conducted to retrieve papers that could identify the barriers and enablers to SAP guideline adherence.

Four electronic databases (CINAHL, EMBASE, PubMed and SCOPUS) were searched for articles that were published in English between January 1998 and December 2018. Search terms included “antibiotic prophylaxis”, “practice guidelines” and “guideline adherence”—a combination of free text and MeSH headings were used where appropriate. The Boolean operators OR and AND were used to combine the search terms. Reference lists of full-text papers that met the eligibility criteria were also hand searched in order to identify relevant studies that may not have appeared through the database search. The search strategy for each database is listed in Additional file 2.

### Inclusion criteria

Articles were included if (a) they were solely related to SAP (without the assessment of adherence to other infection control measures); (b) discussed barriers or enablers to SAP guideline adherence; (c) SAP guidelines were pre-existing before the study was conducted (either through local, institutional guidelines or through the use of national or international guidelines); (d) were in the English language; (e) were original, peer-reviewed articles; and (f) the full-text articles could be sourced.

### Exclusion criteria

Articles were excluded if (a) they were not related to SAP, (b) discussed multiple infection control measures alongside SAP guideline use, (c) did not assess/discuss the factors that influence adherence to SAP guidelines, (d) discussed treatment or therapeutic doses of antibiotics rather than prophylaxis, (e) were audits that solely discussed compliance rates with SAP guidelines without an explanation of the factors that influenced use, (f) discussed perceived barriers or enablers to SAP guideline adherence (rather than reported/factual factors), (g) reviewed how guideline adherence affected infection rates, (h) discussed factors that were based on statistical analyses only and (i) SAP guidelines were developed as part of a study before assessing the effects of interventions that may influence adherence. Additionally, grey literature (e.g. conference papers and theses) as well as review papers were excluded.

### Selection of studies

Database search results were exported to Endnote version X9.2 (Clarivate Analytics), where duplicates were removed. Titles and abstracts were initially screened by a single author (SH), due to the efficiency and acceptability of this process [33], and papers that were potentially relevant based on the eligibility criteria underwent a full-text review. Full-text papers were independently reviewed by two authors (SH and IS). Where discrepancies were found in the outcomes, discussions were made with two authors (VC and JS) until a consensus could be reached.

### Assessment of quality

The Mixed Methods Appraisal Tool (MMAT) was used to assess the quality of included studies [34]. The MMAT is a quality appraisal tool that appraises the methodological quality of qualitative, quantitative (randomised controlled, non-randomised, quantitative descriptive) and mixed methods studies.

Ten papers (21%) were randomly selected and independently assessed by all four authors. Discussions took place where discrepancies were identified until a consensus could be reached. The remaining papers were then independently assessed by one author (SH), due to the acceptability of single author appraisal [33]. Papers were assigned a score based on the percentage of criteria that was met for the relevant study design (i.e. if “Yes” was selected 4 out of 5 times, a score of 80% was given). For papers that were classified as mixed methods studies, 3 sets of criteria were used to determine the final score (using the qualitative study criteria, quantitative study criteria and the mixed methods study criteria). Papers were then categorised into one of three categories—where low quality was considered to be a score between 0 and 40%, medium quality between 41 and 70% and high quality between 71 and 100%.

### Data extraction and analysis

Data was extracted and tabulated. Data retrieved from the papers included a list of reported barriers that prevented adherence to guideline use as well as any enablers (i.e. interventions employed by included studies to promote adherence to SAP guidelines). For articles that described an intervention that was deemed unsuccessful, potential reasons behind the outcome were documented. Data collected on barriers and enablers were mapped to the TDF. Barriers were further categorised under the headings personal or organisational barriers, while enablers were categorised under the interventions that were employed by included studies. Mapping of domains was reviewed by all four authors and agreed upon accordingly.

## Results

### Article selection

**Fig 1.**
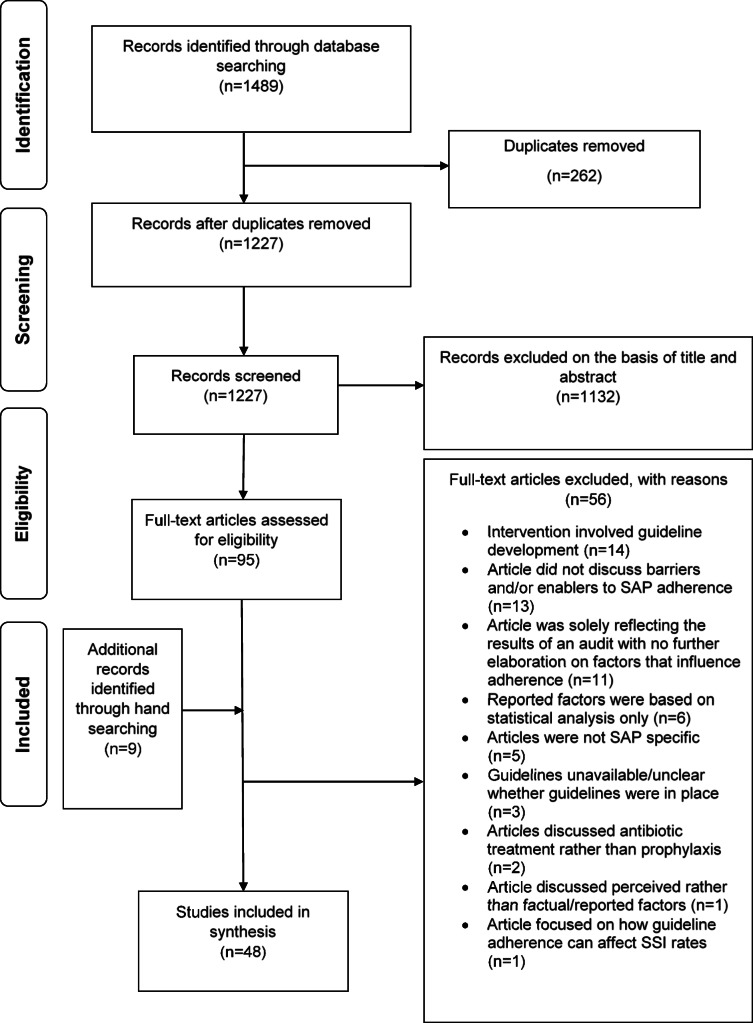
PRISMA flow diagram

A total of 1489 studies were initially retrieved through the database search. After removing duplicated articles (*n* = 262), the title and abstracts of 1227 articles were reviewed. From this, 1132 records were excluded on the basis of title and abstract, resulting in 95 papers with potential for inclusion. The reference list of full-text articles were also hand searched, resulting in a further 9 papers that were reviewed for eligibility. After reviewing the full-text articles, a total of 48 papers were deemed relevant and thus included for synthesis (Fig. 1). A list of articles that were excluded can be found in Additional file 3.

### Characteristics of selected studies

Of the 48 papers included in this review, the majority highlighted enablers (i.e. successful interventions) that permitted SAP guideline adherence (36/48). Six papers solely discussed barriers to guideline adherence, and three papers discussed interventions that were neither a barrier nor an enabler to guideline use while the remaining three papers highlighted factors that both prevented and promoted guideline adherence. Almost half of the studies were conducted in the USA (22/48), three in Australia, two each in Brazil, Canada, France, Italy, Jordan and the UK while the remaining studies were conducted in Belgium, China, Greece, Ireland, Korea, New Zealand, Nigeria, Qatar, South Africa, Spain and Turkey.

### Assessment of quality

All 48 papers were assessed using at least one of the study designs listed in the MMAT [34]. The most common criteria used to assess the quality were questions pertinent to a non-randomised study (36/48), followed by quantitative descriptive studies (4/48) and qualitative studies (3/48). Two papers were classified as randomised control trials, two papers used two different quantitative approaches and one paper used a mixed methods study design and so was assessed accordingly.

Thirty-nine papers were deemed to be of high quality, with 25 papers scoring 80% while the remaining 14 papers received a score of 100%. Of the remaining 9 papers, eight papers were classified as being of medium quality (60% score achieved in 7/8 papers, 70% for 1/8 papers). Only one study was categorised as low quality, receiving a score of 40%; however, we did not exclude any studies on the basis of quality. The results of individual studies can be found in Additional file 4.

### Key findings of individual studies

A summary of the findings can be found in Tables 1, 2, 3 and 4. Table 1 highlights the reported barriers while Table 2 highlights the reported enablers of included studies. Table 3 describes studies that discussed both barriers and enablers, while Table 4 describes studies in which interventions employed were neither a barrier nor enabler to SAP guideline adherence.

**Table 1:** Summary of reported barriers

Author (year) and country	Study design and population	TDF Domain	Description of reported barrier
Al-Azzam et al. (2012) [35] Jordan	Quantitative, descriptive (cross-sectional survey)	Knowledge	**Personal barrier (intrapersonal):** - Lack of guideline knowledge
	Physicians	Environmental context and resources	**Organisational barriers:** - Work flow - Lack of organisational communication - Drug unavailability - Drug cost - Presence of institutional policy (preventing the use of international guidelines – note that authors were determining compliance to international guidelines in this paper)
Bonfait et al. (2010) [36] France	Quantitative, descriptive	Social/Professional role and identity	**Personal barriers (interpersonal):** - Lack of role delegation for prescribing and administering antibiotics
	Orthopaedic surgeons	Knowledge	**Personal barriers (intrapersonal):** - Lack of awareness of guideline content - Antibiotics not administered due to “negligence or oversight”
		Memory, attention and decision processes	
		Environmental context and resources	**Organisational barriers:** - Lack of communication between specialties (anaesthetists and surgeons) at induction - Insufficient staff training - Excessive workload and inappropriate work allocation - Lack of written guidelines - Guidelines present in the wrong place – inaccessible in theatre or on the wards
Broom et al. (2018) [37] Australia	Qualitative	Memory attention and decision processes	**Personal barriers (intrapersonal):** -Forgetfulness - Lack of confidence in ability to protect against adverse consequences/ fear of repercussions (infections) hence extended duration of prophylaxis (“peace of mind”) - Level of experience (junior vs senior)
	Surgeons Anaesthetists	Beliefs about consequences	
		Beliefs about capabilities	
		Emotion	
		Skills	
		Knowledge	
		Social influences	**Organisational barriers:** - Culture of improvisation as the norm rather than guideline adherence - Antibiotic prophylaxis is seen as low priority by staff in theatre especially if competing demands are present
		Environmental context and resources	
Chen et al. (2018) [38] USA	Quantitative descriptive	Knowledge	**Personal barriers (intrapersonal):** - Lack of awareness - Reliance on personal experience to determine practice
		Beliefs about capabilities	
	Paediatric electrophysiologists	Environmental context and resources	**Organisational barriers:** - Presence of institutional guidelines (preventing national guidelines from being used – note that authors were reviewing compliance to national guidelines in this study) - Lack of data present for paediatric population (hence national guidelines not being adhered to)
Madubueze et al. (2015) [39] Nigeria	Quantitative descriptive	Skills	**Personal barriers (intrapersonal):** - Habits that have been picked up during training or practice - Belief that proper aseptic techniques are not being followed on site
	Orthopaedic surgeons	Beliefs about consequences	
		Environmental context and resources	**Organisational barriers:** - Work environment sterility (not considered clean enough hence the extension of antibiotic use)
Tan et al. (2006) [40] Canada	Qualitative	Social/professional role and identity	**Personal barriers (interpersonal):** **Role perception:** *- Shared responsibility:* belief that there is a shared responsibility in administering antibiotics (should be administered by whoever it is most convenient for at the time) *- Individual responsibility*: belief that antibiotic should be administered by nurse or anaesthesiologist *- Resignation*: anaesthesiologists expressed resentment at having to administer antibiotics – was considered external to scope of practice; violation of medical hierarchy
	Anaesthesiologists Surgeons Peri-operative administrators (nurse/anaesthesia administrators) Nurses Pharmacist	Social influences	
		Emotion	
		Environmental context and resources	**Organisational barriers:** - Inherent unpredictability of workflow systems as well as unanticipated changes to workflow - Antibiotic prophylaxis considered as low priority given other competing concerns in theatre - Administration is seen as inconvenient as it disrupts preoperative routine - Lack of verbal communication regarding antibiotics information

**Table 2:** Summary of reported enablers

Author (year) and country	Study design and population	TDF Domain	Description of reported enablers
Brink et al. (2017) [41] South Africa	Quasi-experimental (pre-post)	Behavioural regulation	**Audit and feedback:** **-** Employment of pharmacists to implement an audit and feedback model in relation to adhering to guidelines; providing feedback in written and verbal form - Benchmarking between hospitals and regions via comparative tables and graphs to compare and contrast results
	Pharmacists Surgeons Anaesthetists Theatre and surgical ward nurses Hospital, pharmacy, nursing and theatre managers	Environmental context and resources	**Effective communication:** - Ensuring appropriate communication between multidisciplinary parties
		Skills	
		Social/professional role and identity	**Role delegation:** - Anaesthetists taking responsibility for antibiotic administration
Bryson et al. (2015) [42] UK	Quasi-experimental (pre-post)	Knowledge	**Guideline dissemination:** - Dissemination of information through the use of advertising and educational programs such as announcements on the intranet, displaying posters in theatre, sending out information via email as well as verbally
	Orthopaedic surgeons Anaesthetists	Environmental context and resources	
Cameron et al. (2015) [43] UK	Quasi-experimental (pre-post), Quantitative descriptive	Knowledge	**Guideline dissemination:** - Guideline availability in theatre to help with decision making - Guidelines presented in a simple ‘traffic light system’ format
	Consultant general surgeons Anaesthetists	Environmental context and resources	
Carlès et al. (2006) [44] France	Quasi-experimental (pre-post)	Behavioural regulation	**Support tools:** - Use of personalised SAP kits for patients undergoing surgery that are prepared by pharmacy in advance
	Anaesthesiologists	Environmental context and resources	
Caruso et al. (2017) [45] USA	Quasi-experimental (pre-post)	Social influences	**Multidisciplinary collaboration:** - Teamwork between specialties to develop plans in order to increase SAP adherence
	Paediatric anaesthesiologists Surgeons Infectious disease physicians Pharmacists Quality improvement specialists	Behavioural regulation	**Audit and feedback:** - Monitoring of adherence via compliance reports **Support tools:** - Incorporating antibiotic order set into pre-existing pre-surgical admission routine work **Guideline dissemination:** - Dissemination of dosage information via signs posted in theatre and visual aid pocket cards
		Environmental context and resources	
		Knowledge	
Collier et al. (1998) [46] USA	Quasi-experimental (pre-post)	Knowledge	**Educational services:** - Workshops to inform clinical staff of organisational changes that would be made to improve practice
	Vascular surgeons Anaesthetists Theatre and ward nurses Pharmacists	Environmental context and resources	**Other enablers:** - Rectifying issues as they are identified
Conaty et al. (2018) [47] Ireland	Quasi-experimental (pre-post)	Behavioural regulation	**Audit and feedback:** *- Weekly* audit and feedback sessions (*regular* surveillance)
	Orthopaedic surgeons Anaesthetists Nurses Pharmacists	Knowledge	**Educational services:** - Educational workshops and reminders on appropriate SAP **Guideline dissemination:** - Dissemination of guidelines electronically and through posters displayed in theatre
		Environmental context and resources	
De Almeida et al. (2012) [48] Brazil	Quasi-experimental (pre-post)	Social influences	**Multidisciplinary collaboration:** - Collaboration between an ICU pharmacist and infectious diseases physician when reviewing patients to ensure guidelines are adhered to
	Infectious disease physician Pharmacist Intensivist	Environmental context and resources	**Audit and feedback:** - Continual surveillance through compliance reports disseminated electronically and verbally
Dimopoulou et al. (2016) [49] Greece	Quasi-experimental (pre-post)	Knowledge	**Educational services:** - Workshops regarding appropriate SAP and SSI prevention **Guideline dissemination:** - Distribution of guidelines to clinical team
	Paediatric surgeons, nurses and anaesthetists	Environmental context and resources	
		Social/professional role and identity	**Role delegation:** - Transferring the responsibility of SAP prescribing to the surgeon, whilst administration of antibiotics is to be carried out by the ward nurse or anaesthetist
		Reinforcement	**Local opinion leader:** - Use of a champion or a leader to assist with adhering to guidelines (to reinforce guideline use)
		Social influences	
Garcell et al. (2017) [50] Qatar	Quasi-experimental (pre-post)	Behavioural regulation	**Audit and feedback:** *- Regular* audit and feedback sessions
	Surgical staff	Knowledge	**Educational services:** - Discussions around local SAP policy
		Environmental context and resources	
Haynes et al. (2011) [51] USA	Quasi-experimental (pre-post)	Behavioural regulation	**Support tools:** - Use of a special antibiotic order set with an automated time system to force cessation of antibiotic use once time was exceeded. Removing the prescribing duration from the prescriber
	Physicians	Environmental context and resources	
Hermsen et al. (2008) [52] USA	Quasi-experimental (pre-post)	Behavioural regulation	**Support tools:** - Development and use of a standardised antibiotic order form to provide guidance on antibiotic choice, duration and dose **Educational services:** - Educational sessions to doctors, pharmacists and nurses in regards to the form and the Surgical Infection Prevention Program
	Physicians Pharmacists Nurses	Environmental context and resources	
		Knowledge	
Hincker et al. (2017) [53] USA	Quasi-experimental (pre-post)	Behavioural regulation	**Support tools:** - Modification of EMR system to include decision support tool to guide antibiotic choice and re-dosing interval **Real-time reminders:** - Electronic reminder (on EMR) to indicate when patient should be re-dosed; real-time information
	Anaesthesia staff	Environmental context and resources	
		Reinforcement	
Kao et al. (2010) [54] USA	Staggered cohort	Knowledge	**Educational sessions:** - Lectures to clinical staff - Drawing attention to importance of compliance **Support tools:** - Use of a preoperative checklist and standardised forms to order antibiotics **Other enablers:** - Staggering introduction of interventions to give staff an opportunity to implement change
	Anaesthesia and surgical staff	Environmental context and resources	
		Behavioural regulation	
Kim et al. (2012) [55] Korea	Quasi-experimental (pre-post)	Behavioural regulation	**Audit and feedback**: - Reporting of results to the public as well as to hospitals
	Not specified		
Kritchevsky et al. (2008) [56] USA	Cluster randomised trial	Behavioural regulation	**Support tools:** -Development of prewritten order set for antibiotics -Guidelines, forms and literature reviews shared amongst intervention hospitals -Monthly conferences **Audit and feedback:** -Comparative feedback reports sent to all participating hospitals **Educational services:** - Monthly conferences held to discuss issues and successes experienced by participating intervention hospitals -Meetings held to discuss strategies on how to overcome obstacles related to practice
	Physician assistants Nurse practitioners Pharmacists Surgeons Anaesthesiologists	Environmental context and resources	
		Knowledge	
Lingard et al. (2011) [57] Canada	Quasi-experimental (pre-post)	Behavioural regulation	**Support tools:** - Use of a checklist to prompt antibiotic use and documentation **Effective communication:** - Comprehensive preoperative team briefings: enhanced communication between multidisciplinary staff members
	General surgeons Anaesthesiologists Theatre nurses Technical assistants	Environmental context and resources	
		Skills	
Nair et al. (2010) [58] USA	Quasi-experimental (pre-post)	Environmental context and resources	**Support tools:** - Implementation of AIMS: electronic anaesthesia documentation system **Audit and feedback:** *- Regular* performance reports indicating success rates - Electronic reminders (via email) to document antibiotic information if missing in AIMS **Real-time reminders:** - Real-time electronic feedback and reminders via a decision support system known as SAMs which is linked to AIMS; reminders to carry out specific action
	Anaesthesiologists Nurse anaesthetists	Behavioural regulation	
		Reinforcement	
Nair et al. (2011) [59] USA	Quasi-experimental (pre-post)	Environmental context and resources	**Real-time reminders:** - Use of SAMs: real time alerts to prompt antibiotic administration in a timely fashion. Frequent messages until action taken
	Anaesthesia staff	Behavioural regulation	
		Reinforcement	
O’Reilly et al. (2006) [60] USA	Quasi-experimental (pre-post)	Environmental context and resources	**Support tools:** - Modification of electronic perioperative systems to allow documentation of antibiotic particulars **Audit and feedback:** *- Regular* performance feedback to individual staff members (electronic). Verbal feedback given to staff who are constantly non-compliant - Publication of performance results to serve as a reminder as well as verbal reminders during staff meetings
	Surgeons Nurses Anaesthesia staff	Behavioural regulation	
		Social/professional role and identity	**Role delegation:** - Anaesthetists responsible for administering antibiotics
Ozgun et al. (2010) [61] Turkey	Quasi-experimental (pre-post)	Knowledge	**Educational services:** - Educational sessions held regarding SAP principles as well as what is considered inappropriate use - Data on current practice presented to staff - Discussion of specialty specific issues discussed with surgical team **Guideline dissemination:** - SAP guidelines distributed during staff meetings and displayed throughout hospital
	Surgeons Anaesthetists Nurses	Environmental	
		context and resources	
Parker et al. (2007) [62] USA	Quasi-experimental (pre-post)	Behavioural regulation	**Support tools:** -Use of standardised preoperative antibiotic order forms **Real time reminders:** -Use of an anaesthesia record keeping system to remind anaesthesia provider of appropriate time to administer antibiotics
	Non-cardiac surgeons Anaesthesiologists and anaesthesia care providers Preoperative nursing staff	Environmental context and resources	
		Reinforcement	
		Social/professional role and identity	**Role delegation**: -Delegating role of confirming and administering antibiotic prophylaxis to anaesthesia personnel -Antibiotics sent with patients to theatre in order to assist with its administration before incision
		Knowledge	**Educational sessions:** -Educating staff on antibiotic prophylaxis in order to change attitudes towards use
		Environmental context and resources	
Ribed et al. (2018) [63] Spain	Quasi-experimental (pre-post)	Environmental context and resources	**Support tools:** - Prepopulating SAP information onto the CPOE to assist with correct antibiotic prescribing **Real-time reminders:** - Integrating reminders into clinician’s workflow to modify patient’s prescription based on lab results **Educational services:** - Educational sessions to increase SAP awareness - Pharmacy led training sessions on how to use the CPOE
	Orthopaedic surgeons Nurses Pharmacists	Behavioural regulation	
		Reinforcement	
		Knowledge	
Riggi et al. (2014) [64] USA	Quasi-experimental (pre-post)	Environmental context and resources	**Real-time reminders:** - Use of an automated intraoperative paging system to ensure antibiotics are re-dosed at the appropriate time during lengthy procedures **Educational services:** - Education sessions to anaesthesiology and surgical staff **Audit and feedback:** - Feedback to staff when non-compliance occurred (to individual staff member and chairman of anaesthesiology) **Other enablers:** - Standardisation of SAP protocol (hospital wide)
	Surgical staff Anaesthesia staff	Knowledge	
		Behavioural regulation	
		Reinforcement	
Ritchie et al. (2004) [65] New Zealand	Quasi-experimental (pre-post)	Behavioural regulation	**Real-time reminders:** - Use of a pre-printed sticker on medication chart that included antibiotic particulars (dose, duration and dosing interval) to assist with correct prescribing: information present at point of care – immediate reminder of antibiotic policy
	Anaesthetists Pharmacists		
		Reinforcement	
		Environmental context and resources	
		Social/professional role and identity	**Role delegation:** - Anaesthetists responsible for applying sticker as they administer first dose
Rosenberg et al. (2008) [66] USA	Quasi-experimental (pre-post)	Behavioural regulation	**Support tools:** - “Piggybacking” antibiotic administration verification to surgical time out sheet (preoperative checklist). Acts as a prompt to ensure antibiotics are administered prior to incision
	Anaesthesiologists Theatre nurses	Environmental context and resources	
Schwann et al. (2011) [67] USA	Quasi-experimental (pre-post)	Behavioural regulation	**Real-time reminders:** - Use of a POCEP to provide real-time notifications during a procedure to administer antibiotics: reminder system
	Anaesthesiologists Certified registered nurse anaesthetists Surgeons	Reinforcement	
		Environmental context and resources	
		Social/professional role and identity	**Role delegation:** - Anaesthesiologist or certified registered nurse anaesthetist responsible for validating the POCEP
Shapiro et al. (2018) [68] USA	Quasi-experimental (pre-post)	Environmental context and resources	**Educational services:** - Educational sessions highlighting current recommendations: presentation of current practice highlighting antibiotic usage (transparency of practice)
	Gynaecological surgeons	Knowledge	
Sutherland et al. (2014) [69] USA	Quasi-experimental (pre-post)	Behavioural regulation	**Audit and feedback and multidisciplinary collaboration:** - Multidisciplinary involvement in feedback committee - Discussions/contact made with staff who are constantly non-compliant to review practice
	Anaesthesiologists Surgeons	Social influences	
Telfah et al. (2015) [70] Jordan	Quasi-experimental (pre-post)	Knowledge	**Educational services:** -Revising guidelines to ensure it is comprehensive then providing education to clinical staff to increase awareness -One to one educational sessions and email reminders also provided
	Medical, nursing and pharmacy staff Surgical residents	Environmental context and resources	
		Social/professional role and identity	**Role delegation:** - Assigning a clinical pharmacist to review and evaluate prescribed medications to ensure compliance
		Environmental context and resources	**Other enablers:** -Creation of a theatre satellite pharmacy to allow pharmacists to review and process all orders prior to supply, whilst also restricting access to antibiotics
Wax et al. (2007) [71] USA	Quasi-experimental (pre-post)	Reinforcement	**Real-time reminders:** -Activation of a visual reminder on the AIMS to ensure antibiotics are given before incision
	Anaesthesia care team (certified registered nurse anaesthetist, anaesthesia house staff and attending anaesthesiologist)	Behavioural regulation	
		Environmental context and resources	
Whitman et al. (2008) [72] USA	Quasi-experimental (pre-post)	Social/professional role and identity	**Role delegation:** - Anaesthetists assumed responsibility of antibiotic administration in theatre
	Anaesthetists	Behavioural regulation	**Support tools:** - Use of a preoperative order form in preadmission clinic to ensure antibiotics are charted early **Other enablers:** - Ensuring patients don’t leave hold area until antibiotics are administered
		Environmental context and resources	
Willems et al. (2005) [73] Belgium	Quasi-experimental (pre-post)	Behavioural regulation	**Audit and feedback:** - Highlighting cost to hospital when SAP not adhered to (use of a follow up form to highlight cost of antibiotics when following SAP guidelines vs the antibiotic regimen used by the doctor)
	Physicians		
Zanetti et al. (2003) [74] USA	Randomised control trial	Environmental context and resources	**Real-time reminders:** - Use of an audible alarm (computer generated) to notify staff when antibiotic needs to be re-dosed as well as pop-up notification displaying re-dosing guidelines
	Surgical staff	Behavioural regulation	
		Reinforcement	
Zanotto et al. (2006) [75] Brazil	Quasi-experimental (pre-post)	Behavioural regulation	**Support tools:** - Placing software restrictions on certain antibiotics to limit inappropriate prescribing - Interruption of dispensing if no reason documented electronically to justify extended antibiotic use
	Not specified	Environmental context and resources	
Zhou et al. (2016) [76] China	Quasi-experimental (pre-post)	Social/professional role and identity	**Role delegation:** - Delegating task of reviewing antimicrobial prescribing to clinical pharmacist
	Pharmacists Surgeons Nurses	Knowledge	**Educational services:** - Educational sessions to medical, surgical and nursing staff regarding appropriate SAP
		Environmental context	
		Behavioural regulation	**Audit and feedback:** *- Weekly* performance reports regarding SAP adherence and irrational antibiotic use - Communication between staff when inappropriate prescribing detected

*Abbreviations*: *AIMS* Anaesthesia Information Management System, *CPOE* computerised physician order entry, *EMR* electronic medical record, *POCEP* point-of-care electronic prompt, *SAM* Smart Anaesthesia Manager, *SAP* surgical antimicrobial prophylaxis, *SSI* surgical site infection

**Table 3:** Summary of studies that discussed both barriers and enablers

Author (year) and country	Study design and population	TDF Domain	Description of reported barrier	TDF Domain	Description of reported enabler
Broom et al. (2018) [77] Australia	Qualitative	Social/professional role and identity	**Personal barriers (interpersonal):** **Relationship between surgeon and anaesthetist:** - Poor communication between surgeons and anaesthetist - Lack of task delegation in regards to antibiotic use **Hierarchy within and between surgical and anaesthetist teams:** - Hierarchy affects whether a colleague’s decision would be “challenged”	Skills	**Effective communication:** - Working in a private hospital sector as communication was seen as better. Improved responsibility sharing between surgeon and anaesthetist
	Surgeons Anaesthetists	Social influences		Environmental context and resources	
		Environmental context and resources			
		Beliefs about capabilities	**Personal barrier (intrapersonal):** - Surgeon level of experience influences whether or not they choose to prescribe SAP (junior vs senior staff)		
		Skills			
		Knowledge			
		Environmental context and resources	**Organisational barriers:** - Workflow – especially emergency settings, communication and consultation may not occur. SAP may not be considered a priority - Effect of influential staff members on local cultures of prescribing (again the effect of hierarchy influencing correct SAP use)		
		Social influences			
Giusti et al. (2016) [78] Italy	Mixed methods	Environmental context and resources	**Personal barriers (intrapersonal):** - Disagreement between health care professionals and content in guidelines - Belief that antibiotics listed in guidelines are not efficacious - Individual understanding of the meaning of prophylaxis; poorer understanding meant that antibiotic use was extended as a precautionary measure - Poor knowledge of local hospital data on how SAP is used and the incidence of SSIs - Level of experience: older, more experienced staff more likely to follow personal experience over guidelines	Knowledge	**Guideline dissemination:** - Dissemination of guidelines, particularly when shared and communicated appropriately
	Anaesthesiologists Surgeons Nurse coordinators	Knowledge		Environmental context and resources	
		Beliefs about capabilities		Social influences	**Multidisciplinary collaboration:** - Trust in guideline developers. Multidisciplinary collaboration to develop guidelines
				Beliefs about consequences	**Other enablers:** - Belief that guideline adherence can act as a protective tool if legal action is taken against practitioner
		Environmental context and resources	**External barriers:** - Parental expectation that SAP would be used - Pharmaceutical company pressure in regards to choice of antibiotic **Organisational barriers:** - Availability of hand hygiene facilities – overcrowding of patient rooms during visiting hours can lead to extended prophylaxis		
Nobile et al. (2014) [79] Italy	Quasi-experimental (pre-post), Quantitative descriptive	Environmental context and resources	**Organisational barrier:** - Lack of guideline presence on wards	Social influences	**Multidisciplinary collaboration:** - Collaboration to review and update existing guidelines
	Orthopaedic surgeons			Knowledge	**Educational services:** **-** Educational sessions to explain SSI prevention,
	Nurses Pharmacists			Environmental context and resources	guidelines as well as the correct administration of SAP **Guideline dissemination:** - Development of pocket sized guidelines for quick reference
				Behavioural regulation	**Audit and feedback:** - Feedback given to staff when deviation from practice detected (*regular* monitoring and evaluation of practice)

*Abbreviations*: *SAP* surgical antimicrobial prophylaxis, *SSI* surgical site infection

**Table 4:** Interventions employed that did not influence guideline adherence

Author (year) and country	Study design and population	Intervention	Potential reasons for outcome
Knox and Edye (2016) [80] Australia	Quasi-experimental (pre-post)	Education and increasing awareness without attempting to change practice - Display of SAP guidelines for majority of the surgical procedures in surgical areas—mainly in theatre. Information present included the recommended drug, dose, time and duration - Substantial advertising throughout the hospital site to raise general awareness of appropriate prescribing of antibiotics in all clinical areas	Knox and Edye [80] believe low uptake may be due to cognitive dissonance as the educational interventions used were passive in nature
	Not specified		
Nemeth et al. (2010) [81] USA	Quasi-experimental (pre-post)	- Education of anaesthesia, surgical and nursing staff for a one month period - Modification of pre-operative checklist to include confirmation of timely antibiotic administration	Nemeth et al. [81] believe that results were lower in the post-intervention group due to: (a) Pre-operative verification not being conducted (b) Verification being conducted incorrectly (c) An inappropriate response or lack of response to verification Furthermore, pre-intervention compliance rates were quite high (90%) and sustained effects of intervention could not be observed due to short duration of post-intervention period (5 days)
	Anaesthesia, nursing and surgical staff		
Putnam et al. (2015) [82] USA	Quasi-experimental (pre-post)	- Pre-operative checklist modification to ensure antibiotics are correctly administered - CPOE used so that physicians can order antibiotics from pharmacy at any point prior to procedure - Role delegation—anaesthetists responsible for administering antibiotics - Attachment of guidelines to anaesthesia carts in theatre - Revised guidelines disseminated electronically to all peri-operative staff	Putnam et al. [82] believe that outcomes were poor due to: (a) Little effort in disseminating the CPOE (b) Minimal education being provided on how to use the program (c) Lack of monitoring of CPOE use after implementation (d) Poor dissemination and implementation of the intervention cycles and guidelines
	Paediatric surgeons, anaesthesiologists and peri-operative staff		

*Abbreviations*: *CPOE* computerised physician order entry, *SAP* surgical antimicrobial prophylaxis

### Barriers to guideline adherence

A total of nine papers discussed barriers to SAP guideline adherence (Tables 1 and 3) [35–40, 77–79]. Barriers were often grouped as personal or organisational barriers and were mapped to the following nine TDF domains: knowledge [35–38, 77–79], environmental context and resources [35–40, 77–79], social/professional role and identity [36, 40, 77], memory, attention and decision processes [36, 37], beliefs about consequences [37, 39], beliefs about capabilities [37, 38, 77, 78], emotion [37, 40], skills [37, 39, 77] and social influences [37, 40, 77].

Three papers discussed lack of guideline knowledge or awareness of guideline content as a reason behind poor SAP guideline use [35, 36, 38]. Poor communication between specialties, namely surgical and anaesthetics, also appeared as a recurrent theme in the papers [35, 36, 40]. This lack of communication often meant that tasks relating to antibiotic prescribing and administration were not delegated [36, 77].

### Enablers to guideline adherence

A total of thirty-nine papers discussed enablers that promoted the use of SAP guidelines (Tables 2 and 3) [41–79]. Multiple interventions were developed and incorporated into practice to enhance guideline use. Eight relevant TDF domains were identified: behavioural regulation [41, 44, 45, 47, 50–60, 62–67, 69, 71, 73–76, 79], environmental context and resources [41–54, 56–60, 62–68, 70–72, 74–79], skills [41, 57, 77], social/professional role and identity [41, 49, 60, 62, 65, 67, 70, 72, 76], knowledge [42, 43, 45–47, 49, 50, 52, 54, 56, 61–64, 68, 70, 76, 78, 79], social influences [45, 48, 49, 69, 78, 79], reinforcement [49, 53, 58, 59, 62–65, 67, 71, 74] and beliefs about consequences [78]. A breakdown of the successful interventions that enabled SAP guideline use can be found in Tables 2 and 3.

### Interventions that had minimal effect on guideline adherence

Three papers included interventions that failed to increase the uptake of SAP guidelines [80–82]. Of the three papers, two included educational sessions regarding SAP guidelines [80, 81]. Preoperative checklists were also modified in an attempt to increase guideline use [81, 82].

## Discussion

SAP plays a key role in reducing the rates of SSIs, particularly when used alongside infection control measures such as good surgical technique, the use of hand hygiene products and ensuring patient skin preparation prior to procedure [8, 9, 83]. The appropriate use of clinical practice guidelines in a surgical setting can also contribute to good clinical practice and result in better health outcomes for the patient, whilst reducing exposure to unnecessary interventions [84]. Improving guideline uptake, particularly in regard to ensuring antibiotics are prescribed and administered only for the recommended duration, can help reduce the risk of antimicrobial resistance [9]. However, poor adherence rates to SAP guidelines has been noted [16–19]; thus, the need to determine the barriers and enablers to SAP guideline use.

The TDF has been used to explore factors relating to guideline adherence in a recent qualitative study published by Ierano et al. [85] Themes were mapped to ten domains of the TDF, including knowledge, environmental context and resources, behavioural regulation and emotion [85]. Ierano et al. [85] noted that whilst guidelines were deemed to be of value, practice may differ to guideline recommendations due to a clinician’s perception of gaps in the current evidence, thus resulting in deviations from guidelines. Furthermore, although participants in that study were acutely aware of both local and national guidelines, prescriber autonomy was considered to be of greater importance [85].

We were able to map our findings to 11 TDF domains: knowledge, skills, social/professional role and identity, beliefs about capabilities, beliefs about consequences, reinforcement, memory, attention and decision processes, environmental context and resources, social influences, emotion and behavioural regulation. Barriers were further arranged under personal or organisational barriers, while enablers were further categorised under commonly trialled interventions. We present our findings in light of the TDF domains, highlighting the personal and organisational barriers as well as the interventions that can, and has, enabled the uptake of guidelines.

### Knowledge and environmental context and resources

A lack of knowledge and awareness of guideline content was a frequently mentioned barrier to the adherence of SAP guidelines. Being unaware of current knowledge can result in a patient being managed with information that is no longer relevant, potentially causing patient harm [22]. It is crucial that staff undertake regular educational activities in order to assist in implementation of current evidence-based research. Educational sessions provided to clinical staff, consisting of lectures or workshops, were a popular strategy employed by many studies in an attempt to increase staff knowledge and awareness of guideline content [46, 47, 49, 50, 52, 54, 56, 61–64, 68, 70, 76, 79]. These sessions often involved a review of the importance of SAP, its optimal prescribing and infection prevention. Whilst education plays an important role in transferring knowledge and changing practice, an effective method must be used in order for the information to be retained and acted upon. Effective methods of learning that are most successful ensure that health professionals are actively engaged in the presented content [86]. Interestingly, of the three papers that reported interventions that had minimal effect on guideline uptake, two included a form of education as an intervention [80, 81]. The poor outcome presented in these situations could be a result of the non-specific educational intervention used as well as the passive method used to disseminate information, thus highlighting the need for active methods of education to be utilised in a hospital setting [87, 88].

Guidelines contain the most current evidence based research [15] and can often act as a means of education; however, inaccessibility can act as a hindrance to its use. Guidelines that were inaccessible, in print or electronic form, presented a concern to hospital staff. In some instances, guidelines were completely inaccessible in theatre or on the wards, preventing its use by clinical staff such as surgeons, ward nurses and anaesthesia personnel (anaesthetists and nurse anaesthetists), while in other instances it was hard to locate [36, 79]. Whilst guideline dissemination has been reported as an enabler to SAP guideline adherence, it is important to note that the method of guideline dissemination and presentation also plays a role in determining the likely uptake of content [28]. Guidelines should be presented in a simple, user friendly format that reduces the time required to search for information [28]. An example of a successful intervention present in the review is the use of a “traffic light” poster system which assists staff in determining antibiotic choice, dose and duration of prophylaxis for various gastrointestinal surgical procedures [43]. By providing a colour coded visual reminder in theatre, staff were able to easily refer to guideline information when required, thus resulting in a 2 fold improvement in guideline adherence [43].

Following on, the location of guidelines needs to be considered. In order to make use of SAP guidelines, they need to be present in a location that is readily accessible [28]. The presence of guidelines in operating theatres assists in the uptake of guidelines due to the ease in which it can be accessed [42, 43, 45, 47].

Organisational barriers and the local context can play a role in preventing appropriate adherence to SAP guidelines. The local setting, particularly the culture and practice of staff can influence how SAP guidelines are used. Often, behaviour, beliefs and assumptions are shared by staff in an organisation; however when culture affects performance negatively, staff may become “entrapped” leading to poorer practice [89, 90]. This highlights how settings where improvisation is the norm can heavily impact the extent of guideline incorporation into clinical practice [37]. Furthermore, a lack of agreement of guideline content by health care professionals can result in limited use of guidelines [78], exemplifying the value of including stakeholders in guideline development [78]. Involving key stakeholders in guideline development can increase the likelihood of guidelines being adhered to [79, 85], thus resulting in less variation in how practice is carried out.

Health care professionals often work in settings that are fast-paced, in which there is a high workload and limited resources [91]. Excessive workloads and time constraints place undue pressure on staff, resulting in changes to workflow and preventing staff from providing the best possible care to their patients [36, 92]. One such example is the effect of workflow on the timing of SAP administration. In a qualitative study by Tan et al. [40], participants noted that the unpredictability of workflow systems negatively impacts the timing of antibiotic administration, with the potential to compromise quality standards within hospitals. This is further noted in the study by Al-Azzam et al. [35], in which one third (33%) of participants stated that work flow was a contributing factor to inappropriate timing of SAP administration.

In situations where time is short, health care professionals are more likely to resort to intuitive processes or past practice and experience as a guiding point for their practice rather than rely on and implement recommendations in guidelines [91]. Old habits as well as previous training often overrides the use of evidence-based medicine in these situations, highlighting the need to ensure appropriate time and work allocation to staff [28].

### Skills and beliefs about capabilities

Another barrier to SAP guideline adherence was the reliance on habits picked up during training to guide practice [39]. Overall, 25% of participants in Madubueze et al.’s study [39] indicated that old practices taught in training was a reason for not adhering to SAP guidelines, with nearly half (44%) of participants in the youngest age group surveyed indicating this was also the cause. It was also noted by Broom et al. [37] that junior doctors were more likely to request inappropriate SAP (such as using antibiotics in procedures where it is not required) than senior doctors. Interestingly, the opposite has also been noted. In a mixed methods study by Giusti et al. [78], participants with more than 18 years of practice stated that SAP was determined by personal experience rather than guideline content. In a qualitative study by Ierano et al. [85], it was perceived that younger surgeons were more likely to access guidelines; however, final decisions on antibiotic use was ultimately decided by senior staff. Participants in this study mentioned they did not feel empowered to speak up or challenge senior consultants as it was believed that this would impact their future careers [85].

Effective communication is an important skill required by all health professionals, particularly when practising in a multidisciplinary environment [93]. Effective communication can allow for improved patient safety, greater employee morale and greater flow of information [93]. Thus, enhancing communication between staff such as through comprehensive preoperative team briefings [57] and ensuring consultations take place between staff prior to decision making [41] can help improve guideline adherence.

### Social/professional role and identity and Social influences

Interpersonal barriers were present between staff from multiple specialties, with many presenting between the surgeon and anaesthetist. Lack of communication can lead to SAP mismanagement particularly at induction of anaesthesia, if roles of staff are not clearly determined [35, 36, 40, 77]. Without delegating the task of SAP prescribing and administration, patients may fail to receive the required antibiotic at the relevant time [40]. However, in situations where the role is clear, medical hierarchy can often place a strain or tension on the relationship [77]. Challenging decisions made by senior staff within the same specialty or between specialties is difficult, with many health professionals feeling uncomfortable in doing so [85]. This is especially the case with junior staff, who feel more inclined to accept senior recommendations despite it not aligning with guidelines in an effort to preserve relationships and their career [77, 85].

Role delegation was noted to be an enabler in many studies [41, 49, 60, 62, 65, 67, 70, 72, 76]. Whilst surgeons often took responsibility for prescribing SAP [49, 85], the role of antibiotic administration was delegated to either the anaesthetist or nurse [41, 49, 60, 62, 65, 67, 72]. The correct timing of preoperative administration is important as evidence suggests that administration of antibiotics greater than 120 min prior to incision is associated with a significantly higher risk of SSIs [8]. Thus, it is crucial to ensure that serum and tissue concentrations of antibiotics are adequate at time of incision [14]. As anaesthetists are responsible for administering anaesthesia in theatre as well as other medications [94], many studies found that delegating the role of antibiotic administration to anaesthetists assisted with timely provision of the preoperative dose [41, 60, 62, 72]. In an effort to improve adherence to SAP guidelines, Whitman et al. [72] employed a number of interventions that resulted in an increase in adherence from 55 to 90%. After delegating the role of antibiotic administration to the anaesthetist, this adherence increased further to 95%.

Pharmacists were generally assigned the role of reviewing the appropriateness of prescribed antibiotics to ensure that drug choice and duration of use was suitable [70, 76]. Zhou et al. [76] found that by delegating a pharmacist to review SAP prescribing, adherence to guidelines increased from 83 to 92.2%.

### Emotion and beliefs about consequences

Fear of repercussion and concerns of medical malpractice can drive the overuse of antibiotics, thus resulting in a deviation from standard practice. Although guidelines advocate limiting antibiotic use for surgical prophylaxis where appropriate, the undesirable consequences that can arise from SSI development such as increased morbidity and mortality, hospital readmissions and increased economic burden can result in an extended duration of prophylaxis [37, 78, 85, 95]. Providing additional doses of antibiotics usually adds a perceived layer of comfort for surgeons, thus acting as a line of defence for the surgeon in the situation where a patient develops an infection [37].

### Memory, attention and decision processes, behavioural regulation and reinforcement

To ensure adequate serum and tissue concentrations, intraoperative re-dosing is recommended in SAP guidelines when the duration of a procedure exceeds two half-lives of the administered preoperative drug or if there is excessive blood loss [14]. However, the administration of SAP has been considered low priority during complex and lengthy procedures [37, 40, 77, 85]. Due to the complex procedure at hand, staff may “forget” the need to readminister antibiotics, thus preventing a patient from receiving adequate prophylaxis due to the under-administration of antibiotics [37, 40]. Memory and attention levels can impact the extent to which guidelines are adhered to. Therefore, the use of real-time information, such as electronic prompts and reminders can reinforce guideline information and dramatically increase the level of adherence with SAP guidelines. Nair et al. [58] found that using a real-time feedback and reminder system increased the compliance of timely administration of pre-incisional antibiotics to nearly 100%. In a second study by Nair et al. [59] to determine the impact of electronic reminders on intraoperative re-dosing of antibiotics, the authors found that a real-time reminder system improved the rate of intraoperative re-dosing from 62.5 to ~84%.

The practice of audit and feedback is a popular strategy often implemented in health care settings in order to modify behaviour. As evident by the studies included in this review [41, 45, 47, 48, 50, 55, 56, 58, 60, 64, 69, 73, 76, 79], the coupling of audits with a feedback mechanism generally results in an increased adherence to SAP guidelines. In the study by Sutherland et al. [69], direct physician to physician feedback was used in order to improve adherence to SAP guidelines. Sutherland et al. [69] found that by involving both surgeons and anaesthetists in feedback committees, repeat errors regarding selection and administration of antibiotics could be reduced, thus resulting in an increased adherence to SAP guidelines. It is crucial, however, to ensure that surveillance occurs at regular intervals in order to enhance behaviour change. This provides an opportunity to uncover reasons behind poor guideline adherence whilst allowing for the development of behavioural change strategies. Providing feedback to staff in relation to their practice allows for reflection to take place, thereby resulting in corrective action or motivation to continue performing at the same or a higher level [60]. On a larger scale, presenting results or benchmarking between hospital units or regional districts can lead to competition which can enhance uptake of guidelines at a local setting [41, 85].

Although audit and feedback is seen as a successful means of changing practice, it is important to note this is dependent on factors such as baseline performance of staff and the methods in which feedback is provided [96]. A Cochrane systematic review published in 2012 [96] highlights that, if designed well and used in the right context, audit and feedback can help improve practice. Although the outcomes of this review suggests that the effect of audit and feedback is small to moderate, the effect of feedback can be higher if baseline performance is low, feedback is provided by a supervisor on a regular basis, if feedback is issued in both verbal and written form and an action plan is provided to staff [96].

Papers that reported successful interventions often used a bundle of interventions to change practice. Although the literature contains abundant examples on the benefits of multifaceted interventions in enhancing guideline use [28, 88, 97, 98], Grimshaw and Eccles [99] argue that this may not always be the case. This finding is drawn from a comprehensive systematic review conducted by Grimshaw et al. [100] in which robust statistical techniques were used to make comparisons between intervention types. Grimshaw et al. [100] highlight that previous reviews comparing interventions often used vote-counting to determine effect, whilst providing minimal information regarding effect sizes of interventions. Vote-counting has been used to determine the effectiveness of interventions by comparing the number of positive outcomes to the number of negative outcomes; however, issues may arise by using this technique as it does not provide information on the magnitude of effects [100, 101].

A previous review was conducted by Ng and Chong in 2012 [102] to identify factors that influence a surgeon’s adherence to SAP guidelines. Of note, a lack of awareness and ineffective dissemination of guidelines (such as updating guidelines in a hospital’s handbook without removing old guidelines from theatre) was considered a pivotal reason behind poor guideline use [102, 103]. The review also noted that the use of education and audit and feedback often enhanced guideline adherence [102]. While Ng and Chong’s review [102] specifically highlighted the factors that affect a surgeon’s adherence to guidelines, it did not consider the role of other health professionals in the optimal use of SAP guidelines. In addition, a review by Gouvea et al. in 2015 [104] analysed the adherence rates to SAP guidelines, however, did not undertake an exploration of the factors that resulted in poor adherence.

The strengths of this review include the systematic search of multiple databases through a rigorous search strategy and the evaluation of quality of included studies. The vast majority of papers included were deemed to be of high quality. Through this review, we were able to shed light on the TDF domains that have arisen through the various studies mentioned here, highlighting the factors that affect guideline adherence rates. We focused on identifying the reported barriers and enablers to SAP guideline adherence rather than factors that were perceived or speculated by authors throughout the discussion of their studies. Given the multidisciplinary involvement in SAP management, we did not exclude any health professionals from our review. Through this, we were able to highlight how the practice of surgeons, anaesthetists, nurses and pharmacists contributes to the uptake of guidelines.

Of the limitations of this review, we excluded articles that were in a language other than English which may have limited our results to some extent. We also did not examine the grey literature such as theses or conference abstracts which may discuss the outcomes of quality improvement projects on the effect of SAP guideline adherence. However, we were still able to retrieve a large number of relevant studies from our search. Furthermore, we did not consider studies where guidelines were introduced as part of the intervention. Thus, we were unable to ascertain whether SAP guideline adherence was greater when newly developed guidelines were coupled with other interventions.

## Conclusions

Multiple factors contribute to the suboptimal adherence to SAP guidelines. It is clear that there is a need to identify the factors that may prevent the uptake of guidelines in a local setting, whilst also determining interventions that not only enhance the adherence rates but sustain it for an extended period of time in order to modify practice. Successful studies often employ the use of multiple interventions simultaneously, highlighting the importance of combining different means to change practice. The importance of avoiding passive methods to disseminate information is also clear as the engagement of key stakeholders is crucial to developing change. By understanding the local environment and the nuances that pertain to it, theoretically derived interventions can be developed and implemented, thus increasing the likelihood of adhering to SAP guidelines.

## Supplementary Information

**Additional file 1.** PRISMA 2009 Checklist.

**Additional file 2.** Search strategy.

**Additional file 3.** List of studies excluded from systematic review.

**Additional file 4.** Quality assessment using the Mixed Methods Appraisal Tool (MMAT) Version 2018.

## Data Availability

All data generated or analysed during this study are included in this published article [and its supplementary information files].

## Abbreviations

AIMS: Anaesthesia Information Management System
CDC: Centers for Disease Control and Prevention
CPOE: Computerised physician order entry
EMR: Electronic medical record
MMAT: Mixed method appraisal tool
PRISMA: Preferred Reporting Items for Systematic Reviews and Meta-Analyses
POCEP: Point of care electronic prompt
SAM: Smart Anaesthesia Manager
SAP: Surgical antimicrobial prophylaxis
SSIs: Surgical site infections
WHO: World Health Organization
