# Supplementary material for: Factors that influence adherence to surgical antimicrobial prophylaxis (SAP) guidelines: a systematic review
Source: Syst Rev. 2021 Jan 16;10:29. doi: 10.1186/s13643-021-01577-w (PMC7811740; doi:10.1186/s13643-021-01577-w)
Supplement: Supplementary file 2 — Additional file 2. Search strategy. [file 13643_2021_1577_MOESM2_ESM.docx]

**Additional file 2. Search strategy**

| **Database** | **Search terms** | **Filter** |
| --- | --- | --- |
| **CINAHL** | ((MH "Antibiotic Prophylaxis") OR "antibiotic prophylaxis" OR "antimicrobial prophylaxis" OR "surgical antibiotic prophylaxis" OR "perioperative antibiotic prophylaxis" OR "preoperative antibiotic prophylaxis" OR "antibiotic prophylaxis in surgery" OR "antimicrobial prophylaxis in surgery" OR "surgical antimicrobial prophylaxis" OR "surgery antibiotic prophylaxis" OR "surgical prophylaxis" AND (MH "Practice Guidelines") AND (MH "Guideline Adherence") | - Academic Journals - English Language - 1998-2018 - Sorted by relevance |
| **EMBASE** | ('antibiotic prophylaxis'/exp OR 'antibiotic prophylaxis in surgery' OR 'antimicrobial prophylaxis' OR 'antimicrobial prophylaxis in surgery' OR 'perioperative antibiotic prophylaxis' OR 'preoperative antibiotic prophylaxis' OR 'surgery antibiotic prophylaxis' OR 'surgical antibiotic prophylaxis' OR 'surgical antimicrobial prophylaxis' OR 'surgical prophylaxis') AND ('practice guideline'/exp) AND ('protocol compliance'/exp) | - English Language - 1998-2018 - Articles and articles in press - Sorted by relevance |
| **PubMed** | (((((((((((("Antibiotic Prophylaxis"[Mesh]) OR antimicrobial prophylaxis) OR antibiotic prophylaxis in surgery) OR antimicrobial prophylaxis in surgery) OR perioperative antibiotic prophylaxis) OR preoperative antibiotic prophylaxis) OR surgical prophylaxis) OR surgery antibiotic prophylaxis) OR surgical antimicrobial prophylaxis) OR surgical antibiotic prophylaxis)) AND "Practice Guidelines as Topic"[Mesh]) AND "Guideline Adherence"[Mesh] | - Best match - 1998-2018 - English Language - Humans |
| **SCOPUS** | ( ( TITLE-ABS-KEY ( "antibiotic prophylaxis" )  OR  TITLE-ABS-KEY ( "antibiotic prophylaxis in surgery" )  OR  TITLE-ABS-KEY ( "antimicrobial prophylaxis" )  OR  TITLE-ABS-KEY ( "antimicrobial prophylaxis in surgery" )  OR  TITLE-ABS-KEY ( "perioperative antibiotic prophylaxis" )  OR  TITLE-ABS-KEY ( "preoperative antibiotic prophylaxis" )  OR  TITLE-ABS-KEY ( "surgery antibiotic prophylaxis" )  OR  TITLE-ABS-KEY ( "surgical antibiotic prophylaxis" )  OR  TITLE-ABS-KEY ( "surgical antimicrobial prophylaxis" )  OR  TITLE-ABS-KEY ( "surgical prophylaxis" ) ) )  AND  ( ( TITLE-ABS-KEY ( "clinical guidelines" )  OR  TITLE-ABS-KEY ( "clinical practice guidelines" )  OR  TITLE-ABS-KEY ( "practice guidelines" )  OR  TITLE-ABS-KEY ( guidelines ) ) )  AND  ( ( TITLE-ABS-KEY ( "guideline adherence" )  OR  TITLE-ABS-KEY ( "guideline compliance" )  OR  TITLE-ABS-KEY ( "guideline implementation" )  OR  TITLE-ABS-KEY ( "protocol compliance" ) ) ) | - English Language - 1998-2018 - Articles - Sort by relevance |
