# Supplementary material for: Factors that influence adherence to surgical antimicrobial prophylaxis (SAP) guidelines: a systematic review
Source: Syst Rev. 2021 Jan 16;10:29. doi: 10.1186/s13643-021-01577-w (PMC7811740; doi:10.1186/s13643-021-01577-w)
Supplement: Supplementary file 3 — Additional file 3. List of studies excluded from systematic review. [file 13643_2021_1577_MOESM3_ESM.docx]

**Additional file 3. Full text articles excluded (with reasons)**

| **Reason** | **Reference** |
| --- | --- |
| 1. **Intervention involved guideline development** | 1. So JP, Aleem IS, Tsang DS, Matlow AG, Wright JG. Increasing Compliance With an Antibiotic Prophylaxis Guideline to Prevent Pediatric Surgical Site Infection: Before and After Study. Ann Surg. 2015;262(2):403-8. Epub 2014/11/26. doi: 10.1097/sla.0000000000000934. PubMed PMID: 25423065. |
|  | 1. Yang Z, Zhao P, Wang J, Tong L, Cao J, Tian Y, et al. DRUGS system enhancing adherence of Chinese surgeons to antibiotic use guidelines during perioperative period. PLoS One. 2014;9(8):e102226. Epub 2014/08/26. doi: 10.1371/journal.pone.0102226. PubMed PMID: 25148306; PubMed Central PMCID: PMCPMC4141742. |
|  | 1. Capuano A, Noviello S, Avolio A, Mazzeo F, Ianniello F, Rinaldi B, et al. Antibiotic prophylaxis in surgery: an observational prospective study conducted in a large teaching hospital in Naples. J Chemother. 2006;18(3):293-7. Epub 2006/11/30. doi: 10.1179/joc.2006.18.3.293. PubMed PMID: 17129840. |
|  | 1. Takahashi Y, Takesue Y, Nakajima K, Ichiki K, Wada Y, Tsuchida T, et al. Implementation of a hospital-wide project for appropriate antimicrobial prophylaxis. J Infect Chemother. 2010;16(6):418-23. Epub 2010/06/16. doi: 10.1007/s10156-010-0078-0. PubMed PMID: 20549286. |
|  | 1. Pons-Busom M, Aguas-Compaired M, Delas J, Eguileor-Partearroyo B. Compliance with local guidelines for antibiotic prophylaxis in surgery. Infect Control Hosp Epidemiol. 2004;25(4):308-12. Epub 2004/04/28. doi: 10.1086/502397. PubMed PMID: 15108728. |
|  | 1. Taylor GM. An audit of the implementation of guidelines to reduce wound infection following caesarean section. Health Bull (Edinb). 2000;58(1):38-44. Epub 2003/06/20. PubMed PMID: 12813851. |
|  | 1. McCahill LE, Ahern JW, Gruppi LA, Limanek J, Dion GA, Sussman JA, et al. Enhancing compliance with Medicare guidelines for surgical infection prevention: experience with a cross-disciplinary quality improvement team. Arch Surg. 2007;142(4):355-61. Epub 2007/04/18. doi: 10.1001/archsurg.142.4.355. PubMed PMID: 17438170. |
|  | 1. Alerany C, Campany D, Monterde J, Semeraro C. Impact of local guidelines and an integrated dispensing system on antibiotic prophylaxis quality in a surgical centre. J Hosp Infect. 2005;60(2):111-7. doi: 10.1016/j.jhin.2004.07.022. |
|  | 1. Murray MT, Corda R, Turcotte R, Bacha E, Saiman L, Krishnamurthy G. Implementing a standardized perioperative antibiotic prophylaxis protocol for neonates undergoing cardiac surgery. Ann Thorac Surg. 2014;98(3):927-33. doi: 10.1016/j.athoracsur.2014.04.090. |
|  | 1. Ciofi degli Atti M, Alegiani SS, Raschetti R, Arace P, Giusti A, Spiazzi R, et al. A collaborative intervention to improve surgical antibiotic prophylaxis in children: results from a prospective multicenter study. Eur J Clin Pharmacol. 2017;73(9):1141-7. doi: 10.1007/s00228-017-2270-y. |
|  | 1. Fahy BG, Bowe EA, Conigliaro J. Perioperative antibiotic process improvement reaps rewards. Am J Med Qual. 2011;26(3):185-92. doi: 10.1177/1062860610382133. |
|  | 1. Burnett KM, Scott MG, Kearney PM, Humphreys WG, McMillen RM. The identification of barriers preventing the successful implementation of a surgical prophylaxis protocol. Pharm World Sci. 2002;24(5):182-7. doi: 10.1023/A:1020565000571. |
|  | 1. Kanter G, Connelly NR, Fitzgerald J. A system and process redesign to improve perioperative antibiotic administration. Anesth Analg. 2006;103(6):1517-21. Epub 2006/11/24. doi: 10.1213/01.ane.0000221442.30952.83. PubMed PMID: 17122232. |
|  | 1. Burkitt KH, Mor MK, Jain R, Kruszewski MS, McCray EE, Moreland ME, et al. Toyota production system quality improvement initiative improves perioperative antibiotic therapy. Am J Manag Care. 2009;15(9):633-42. Epub 2009/09/15. PubMed PMID: 19747028. |
| 1. **Article did not discuss barriers and/or enablers to SAP adherence** | 1. Giordano M, Squillace L, Pavia M. Appropriateness of Surgical Antibiotic Prophylaxis in Pediatric Patients in Italy. Infect Control Hosp Epidemiol. 2017;38(7):823-31. Epub 2017/06/06. doi: 10.1017/ice.2017.79. PubMed PMID: 28580893. |
|  | 1. Kacelnik O, Alberg T, Mjaland O, Eriksen H, Skjeldestad FE. Guidelines for antibiotic prophylaxis of cholecystectomies in Norwegian hospitals. Surg Infect (Larchmt). 2013;14(2):188-91. Epub 2013/03/28. doi: 10.1089/sur.2012.015. PubMed PMID: 23530809. |
|  | 1. Hauck RM, Nogan S. The use of prophylactic antibiotics in plastic surgery: update in 2010. Ann Plast Surg. 2013;70(1):91-7. Epub 2011/06/02. doi: 10.1097/SAP.0b013e31821e8f9a. PubMed PMID: 21629059. |
|  | 1. Bae-Harboe YS, Liang CA. Perioperative antibiotic use of dermatologic surgeons in 2012. Dermatol Surg. 2013;39(11):1592-601. Epub 2013/07/20. doi: 10.1111/dsu.12272. PubMed PMID: 23865410. |
|  | 1. Erduran M, Akseki D, Arac S. Surgical practices in total knee arthroplasty in Turkey. Acta Orthop Traumatol Turc. 2012;46(4):255-61. Epub 2012/09/07. PubMed PMID: 22951756. |
|  | 1. Haydon TP, Presneill JJ, Robertson MS. Antibiotic prophylaxis for cardiac surgery in Australia. Med J Aust. 2010;192(3):141-3. Epub 2010/02/04. PubMed PMID: 20121681 |
|  | 1. Schell JA, Bynum CG, Fortune GJ, Laiben GR, Chang YJ, Pirner JA. Perioperative antibiotics in nonemergency bowel surgery: a quality improvement project. South Med J. 1998;91(10):900-8. Epub 1998/10/24. doi: 10.1097/00007611-199810000-00002. PubMed PMID: 9786283. |
|  | 1. Alexiou VG, Ierodiakonou V, Peppas G, Falagas ME. Antimicrobial prophylaxis in surgery: An international survey. Surg Infect (Larchmt). 2010;11(4):343-8. doi: 10.1089/sur.2009.023. |
|  | 1. Bausch K, Roth JA, Seifert HH, Widmer AF. Overuse of antimicrobial prophylaxis in low-risk patients undergoing transurethral resection of the prostate. Swiss Med Wkly. 2018;128(7-8). doi: 10.4414/smw.2018.14594. |
|  | 1. Aiken AM, Haddow JB, Symons NRA, Kaptanis S, Katz-Summercorn AC, Debnath D, et al. Use of antibiotic prophylaxis in elective inguinal hernia repair in adults in London and south-east England: A cross-sectional survey. Hernia. 2013;17(5):657-64. doi: 10.1007/s10029-013-1061-3. |
|  | 1. Sekimoto M, Imanaka Y, Evans E, Ishizaki T, Hirose M, Hayashida K, et al. Practice variation in perioperative antibiotic use in Japan. Int J Qual Health Care. 2004;16(5):367-73. doi: 10.1093/intqhc/mzh066. |
|  | 1. Murri R, de Belvis AG, Fantoni M, Tanzariello M, Parente P, Marventano S, et al. Impact of antibiotic stewardship on perioperative antimicrobial prophylaxis. Int J Qual Health Care. 2016;28(4):502-7. doi: 10.1093/intqhc/mzw055. |
|  | 1. De Beer J, Petruccelli D, Rotstein C, Weening B, Royston K, Winemaker M. Antibiotic prophylaxis for total joint replacement surgery: Results of a survey of Canadian orthopedic surgeons. Can J Surg. 2009;52(6):E229-E34. |
| 1. **Article was solely reflecting the results of an audit with no further elaboration on factors that influence adherence** | 1. Testa M, Stillo M, Giacomelli S, Scoffone S, Argentero PA, Farina EC, et al. Appropriate use of antimicrobial prophylaxis: an observational study in 21 surgical wards. BMC Surg. 2015;15:63. Epub 2015/05/15. doi: 10.1186/s12893-015-0048-7. PubMed PMID: 25968324; PubMed Central PMCID: PMCPMC4434534. |
|  | 1. Hohmann C, Eickhoff C, Radziwill R, Schulz M. Adherence to guidelines for antibiotic prophylaxis in surgery patients in German hospitals: a multicentre evaluation involving pharmacy interns. Infection. 2012;40(2):131-7. Epub 2011/10/18. doi: 10.1007/s15010-011-0204-7. PubMed PMID: 22002734. |
|  | 1. Rangel SJ, Fung M, Graham DA, Ma L, Nelson CP, Sandora TJ. Recent trends in the use of antibiotic prophylaxis in pediatric surgery. J Pediatr Surg. 2011;46(2):366-71. Epub 2011/02/05. doi: 10.1016/j.jpedsurg.2010.11.016. PubMed PMID: 21292089. |
|  | 1. Bedouch P, Labarere J, Chirpaz E, Allenet B, Lepape A, Fourny M, et al. Compliance with guidelines on antibiotic prophylaxis in total hip replacement surgery: results of a retrospective study of 416 patients in a teaching hospital. Infect Control Hosp Epidemiol. 2004;25(4):302-7. Epub 2004/04/28. doi: 10.1086/502396. PubMed PMID: 15108727. |
|  | 1. Abdel-Aziz A, El-Menyar A, Al-Thani H, Zarour A, Parchani A, Asim M, et al. Adherence of surgeons to antimicrobial prophylaxis guidelines in a tertiary general hospital in a rapidly developing country. Adv Pharmacol Sci. 2013;2013. doi: 10.1155/2013/842593. |
|  | 1. El Hassan M, Elnour AA, Farah FH, Shehab A, Al Kalbani NM, Asim S, et al. Clinical pharmacists’ review of surgical antimicrobial prophylaxis in a tertiary hospital in Abu Dhabi. Int J Clin Pharm. 2014;37(1):18-22. doi: 10.1007/s11096-014-0045-4. |
|  | 1. Bull AL, Worth LJ, Spelman T, Richards MJ. Antibiotic Prescribing Practices for Prevention of Surgical Site Infections in Australia: Increased Uptake of National Guidelines after Surveillance and Reporting and Impact on Infection Rates. Surg Infect (Larchmt). 2017;18(7):834-40. doi: 10.1089/sur.2017.119. |
|  | 1. Alemkere G. Antibiotic usage in surgical prophylaxis: A prospective observational study in the surgical ward of Nekemte referral hospital. PLoS One. 2018;13(9). doi: 10.1371/journal.pone.0203523. |
|  | 1. Agrawal M, Sharma PK, Dhaneria SP. Clinical practice audit of perioperative antimicrobial prophylaxis in a tertiary care hospital: Do routine academic activities improve adherence to practice guidelines? Rev Recent Clin Trials. 2017;12(1):59-64. doi: 10.2174/1574887111666160926103104. |
|  | 1. Boriboonhirunsarn D, Lauwahutanont P, Kaewmanee K, Hangsubcharoen M, Uppagan R, Makanantakosol S. Usage of prophylactic antibiotics in uncomplicated gynecologic abdominal surgery in Siriraj Hospital. J Med Assoc Thai. 2007;90(6):1068-73. |
|  | 1. Uppendahl L, Chiles C, Shields S, Dong F, Kraft E, Duong J, et al. Appropriate Use of Prophylactic Antibiotic Agents in Gynecologic Surgeries at a Midwestern Teaching Hospital. Surg Infect (Larchmt). 2018;19(4):397-402. doi: 10.1089/sur.2017.247. |
| 1. **Reported factors were based on statistical analysis only** | 1. Imai-Kamata S, Fushimi K. Factors associated with adherence to prophylactic antibiotic therapy for elective general surgeries in Japan. Int J Qual Health Care. 2011;23(2):167-72. Epub 2011/01/15. doi: 10.1093/intqhc/mzq080. PubMed PMID: 21233110. |
|  | 1. Andrajati R, Vlcek J, Kolar M, Pipalova R. Survey of surgical antimicrobial prophylaxis in czech republic. Pharm World Sci. 2005;27(6):436-41. Epub 2005/12/13. doi: 10.1007/s11096-005-5971-8. PubMed PMID: 16341950. |
|  | 1. Knox MC, Edye M. Adherence to surgical antibiotic prophylaxis guidelines in New South Wales, Australia: Identifying deficiencies and regression analysis of contributing factors. Surg Infect (Larchmt). 2016;17(2):203-9. doi: 10.1089/sur.2015.195. |
|  | 1. Abdel Jalil MH, Abu Hammour K, Alsous M, Hadadden R, Awad W, Bakri F, et al. Noncompliance with surgical antimicrobial prophylaxis guidelines: A Jordanian experience in cesarean deliveries. Am J Infect Control. 2018;46(1):14-9. doi: 10.1016/j.ajic.2017.06.033. |
|  | 1. Schmitt C, Lacerda RA, Turrini RNT, Padoveze MC. Improving compliance with surgical antibiotic prophylaxis guidelines: A multicenter evaluation. Am J Infect Control. 2017;45(10):1111-5. doi: 10.1016/j.ajic.2017.05.004. |
|  | 1. Ukawa N, Tanaka M, Morishima T, Imanaka Y. Organizational culture affecting quality of care: Guideline adherence in perioperative antibiotic use. Int J Qual Health Care. 2015;27(1):37-45. doi: 10.1093/intqhc/mzu091. |
| 1. **Articles were not SAP specific** | 1. Toor AA, Farooka MW, Ayyaz M, Sarwar H, Malik AA, Shabbir F. Pre-operative antibiotic use reduces surgical site infection. J Pak Med Assoc. 2015;65(7):733-6. Epub 2015/07/15. PubMed PMID: 26160082. |
|  | 1. Ayub Khan MN, Verstegen DML, Bhatti ABH, Dolmans D, van Mook WNA. Factors hindering the implementation of surgical site infection control guidelines in the operating rooms of low-income countries: a mixed-method study. Eur J Clin Microbiol Infect Dis. 2018;37(10):1923-9. Epub 2018/08/12. doi: 10.1007/s10096-018-3327-2. PubMed PMID: 30097753. |
|  | 1. Barchitta M, Matranga D, Quattrocchi A, Bellocchi P, Ruffino M, Basile G, et al. Prevalence of surgical site infections before and after the implementation of a multimodal infection control programme. J Antimicrob Chemother. 2012;67(3):749-55. doi: 10.1093/jac/dkr505. |
|  | 1. Meeks DW, Lally KP, Carrick MM, Lew DF, Thomas EJ, Doyle PD, et al. Compliance with guidelines to prevent surgical site infections: As simple as 1-2-3? Am J Surg. 2011;201(1):76-83. doi: 10.1016/j.amjsurg.2009.07.050. |
|  | 1. Shah N, Castro-Sánchez E, Charani E, Drumright LN, Holmes AH. Towards changing healthcare workers' behaviour: A qualitative study exploring non-compliance through appraisals of infection prevention and control practices. J Hosp Infect. 2015;90(2):126-34. doi: 10.1016/j.jhin.2015.01.023. |
| 1. **Guidelines unavailable/unclear whether guidelines were in place** | 1. Musmar SM, Ba'Ba H, Owais A. Adherence to guidelines of antibiotic prophylactic use in surgery: A prospective cohort study in North West Bank, Palestine. BMC Surg. 2014;14(1). doi: 10.1186/1471-2482-14-69. |
|  | 1. Saied T, Hafez SF, Kandeel A, El-kholy A, Ismail G, Aboushady M, et al. Antimicrobial stewardship to optimize the use of antimicrobials for surgical prophylaxis in Egypt: A multicenter pilot intervention study. Am J Infect Control. 2015;43(11):e67-71. Epub 2015/09/01. doi: 10.1016/j.ajic.2015.07.004. PubMed PMID: 26315059. |
|  | 1. St Jacques P, Sanders N, Patel N, Talbot TR, Deshpande JK, Higgins M. Improving timely surgical antibiotic prophylaxis redosing administration using computerized record prompts. Surg Infect (Larchmt). 2005;6(2):215-21. Epub 2005/09/01. doi: 10.1089/sur.2005.6.215. PubMed PMID: 16128628. |
| 1. **Articles discussed antibiotic treatment rather than prophylaxis** | 1. Taleb M, Nardi N, Arnaud A, Costet N, Donnio PY, Engrand C, et al. Simplification of first-line antibacterial regimen for complicated appendicitis in children is associated with better adherence to guidelines and reduced use of antibiotics. Int J Antimicrob Agents. 2018;52(2):293-6. Epub 2018/04/22. doi: 10.1016/j.ijantimicag.2018.04.010. PubMed PMID: 29678565. |
|  | 1. Gorecki W, Grochowska E, Krysta M, Wojciechowski P, Taczanowska A, Stanek B. A prospective comparison of antibiotic usage in pediatric surgical patients: the safety, advantage, and effectiveness of the Surgical Infection Society guidelines versus a common practice. J Pediatr Surg. 2002;37(10):1430-4. Epub 2002/10/16. doi: 10.1053/jpsu.2002.35406. PubMed PMID: 12378448. |
| 1. **Article discussed perceived rather than factual/reported factors** | 1. van Kasteren ME, Kullberg BJ, de Boer AS, Mintjes-de Groot J, Gyssens IC. Adherence to local hospital guidelines for surgical antimicrobial prophylaxis: a multicentre audit in Dutch hospitals. J Antimicrob Chemother. 2003;51(6):1389-96. Epub 2003/05/15. doi: 10.1093/jac/dkg264. PubMed PMID: 12746377. |
| 1. **Article focused on how guideline adherence can affect SSI rates** | 1. Manniën J, van Kasteren MEE, Nagelkerke NJ, Gyssens IC, Kullberg BJ, Wille JC, et al. Effect of Optimized Antibiotic Prophylaxis on the Incidence of Surgical Site Infection. Infect Control Hosp Epidemiol. 2006;27(12):1340-6. Epub 2017/04/07. doi: 10.1017/S0195941700075275. |
