# Supplementary material for: Factors that influence adherence to surgical antimicrobial prophylaxis (SAP) guidelines: a systematic review
Source: Syst Rev. 2021 Jan 16;10:29. doi: 10.1186/s13643-021-01577-w (PMC7811740; doi:10.1186/s13643-021-01577-w)
Supplement: Supplementary file 4 — Additional file 4. Quality assessment using the Mixed Methods Appraisal Tool (MMAT) Version 2018. [file 13643_2021_1577_MOESM4_ESM.docx]

**Additional file 4. MMAT Version 2018**

**
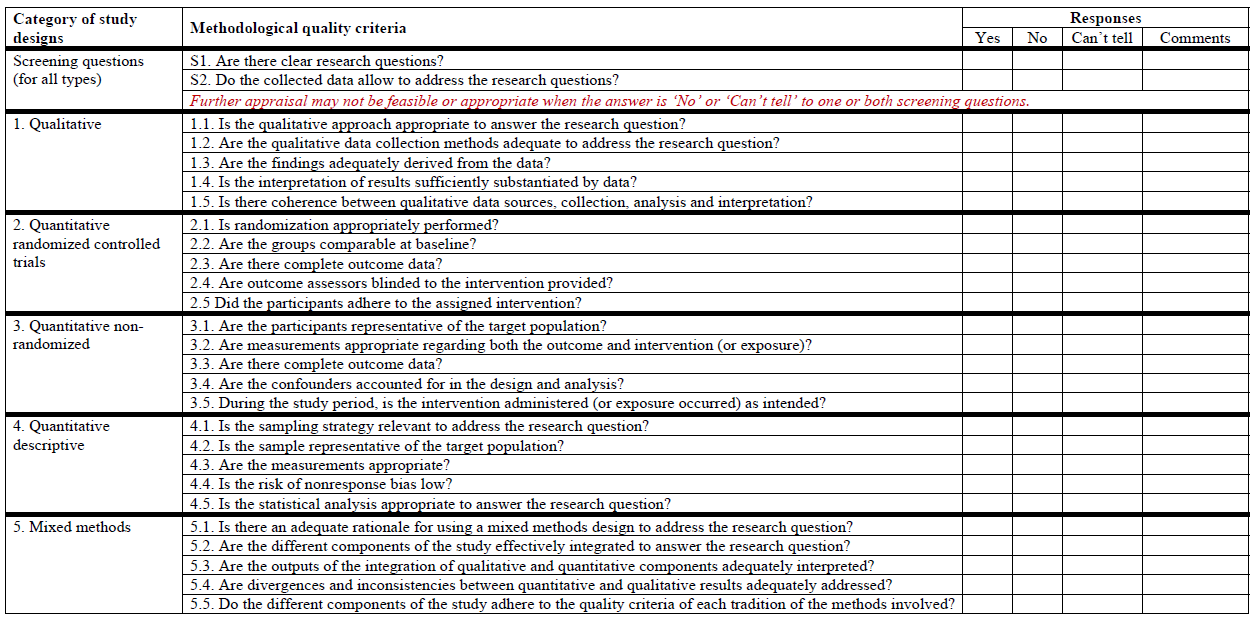
(Quality assessment found below)**

|  | **Qualitative studies** | | | | | **Randomised controlled trials** | | | | | **Non-randomised studies** | | | | | **Quantitative descriptive studies** | | | | | **Mixed methods studies** | | | | |  |
| --- | --- | --- | --- | --- | --- | --- | --- | --- | --- | --- | --- | --- | --- | --- | --- | --- | --- | --- | --- | --- | --- | --- | --- | --- | --- | --- |
| **First Author (Year)** | **1.1** | **1.2** | **1.3** | **1.4** | **1.5** | **2.1** | **2.2** | **2.3** | **2.4** | **2.5** | **3.1** | **3.2** | **3.3** | **3.4** | **3.5** | **4.1** | **4.2** | **4.3** | **4.4** | **4.5** | **5.1** | **5.2** | **5.3** | **5.4** | **5.5** | **Score**  **(%)** |
| Al-Azzam (2012)  [36] |  |  |  |  |  |  |  |  |  |  |  |  |  |  |  | Yes | Yes | Yes | Yes | Yes |  |  |  |  |  | 100 |
| Bonfait (2010)  [37] |  |  |  |  |  |  |  |  |  |  |  |  |  |  |  | Can’t tell | Yes | Yes | Can't tell | Yes |  |  |  |  |  | 60 |
| Brink (2017)  [45] |  |  |  |  |  |  |  |  |  |  | Yes | Yes | Yes | No | Yes |  |  |  |  |  |  |  |  |  |  | 80 |
| Broom  (2018)  [38] | Yes | Yes | Yes | Yes | Yes |  |  |  |  |  |  |  |  |  |  |  |  |  |  |  |  |  |  |  |  | 100 |
| Broom  (2018)  [39] | Yes | Yes | Yes | Yes | Yes |  |  |  |  |  |  |  |  |  |  |  |  |  |  |  |  |  |  |  |  | 100 |
| Bryson (2015)  [46] |  |  |  |  |  |  |  |  |  |  | Yes | Yes | Yes | Can't tell | Yes |  |  |  |  |  |  |  |  |  |  | 80 |
| Cameron  (2015)  [47] |  |  |  |  |  |  |  |  |  |  | Yes | Yes | Yes | Can't tell | Yes | Yes | Yes | Yes | Can't tell | Yes |  |  |  |  |  | 80 |
| Carles  (2006)  [48] |  |  |  |  |  |  |  |  |  |  | Yes | Yes | Yes | Yes | Yes |  |  |  |  |  |  |  |  |  |  | 100 |
| Caruso  (2017)  [49] |  |  |  |  |  |  |  |  |  |  | Yes | Yes | Can't tell | Yes | Yes |  |  |  |  |  |  |  |  |  |  | 80 |
| Chen (2018)  [40] |  |  |  |  |  |  |  |  |  |  |  |  |  |  |  | Yes | Can't tell | Yes | No | Yes |  |  |  |  |  | 60 |
| Collier (1998)  [50] |  |  |  |  |  |  |  |  |  |  | Yes | Yes | Yes | Can't  tell | Yes |  |  |  |  |  |  |  |  |  |  | 80 |
| Conaty (2018)  [51] |  |  |  |  |  |  |  |  |  |  | Yes | Yes | Yes | Can't tell | Yes |  |  |  |  |  |  |  |  |  |  | 80 |
| De Almeida  (2012)  [52] |  |  |  |  |  |  |  |  |  |  | Yes | Yes | Yes | Can't tell | Yes |  |  |  |  |  |  |  |  |  |  | 80 |
| Dimopo-ulou  (2016)  [53] |  |  |  |  |  |  |  |  |  |  | Yes | Yes | Yes | Yes | Yes |  |  |  |  |  |  |  |  |  |  | 100 |
| Garcell  (2017)  [54] |  |  |  |  |  |  |  |  |  |  | Yes | Yes | Yes | Can't tell | Yes |  |  |  |  |  |  |  |  |  |  | 80 |
| Giusti  (2016)  [41] | Yes | Yes | Yes | Yes | Yes |  |  |  |  |  |  |  |  |  |  | Yes | Yes | Yes | Yes | Yes | Yes | Yes | Yes | Yes | Yes | 100 |
| Haynes  (2011)  [55] |  |  |  |  |  |  |  |  |  |  | Yes | Yes | Yes | Can't tell | Yes |  |  |  |  |  |  |  |  |  |  | 80 |
| Hermsen  2008  [56] |  |  |  |  |  |  |  |  |  |  | Yes | Yes | No | No | Yes |  |  |  |  |  |  |  |  |  |  | 60 |
| Hincker  (2008)  [57] |  |  |  |  |  |  |  |  |  |  | Yes | Yes | Yes | Yes | Yes |  |  |  |  |  |  |  |  |  |  | 100 |
| Kao  (2010)  [58] |  |  |  |  |  |  |  |  |  |  | Yes | Yes | Yes | Yes | Yes |  |  |  |  |  |  |  |  |  |  | 100 |
| Kim  (2012)  [59] |  |  |  |  |  |  |  |  |  |  | Yes | Yes | Yes | No | Yes |  |  |  |  |  |  |  |  |  |  | 80 |
| Knox  (2016)  [81] |  |  |  |  |  |  |  |  |  |  | Can't tell | Yes | Yes | Can't tell | Yes |  |  |  |  |  |  |  |  |  |  | 60 |
| Kritchev  -sky  (2008)  [60] |  |  |  |  |  | Yes | Yes | Yes | Can't tell | Yes |  |  |  |  |  |  |  |  |  |  |  |  |  |  |  | 80 |
| Lingard (2011)  [61] |  |  |  |  |  |  |  |  |  |  | Yes | Yes | No | Can't tell | Yes |  |  |  |  |  |  |  |  |  |  | 60 |
| Madubu  -eze  (2015)  [42] |  |  |  |  |  |  |  |  |  |  |  |  |  |  |  | Yes | Yes | Yes | Can't tell | Yes |  |  |  |  |  | 80 |
| Nair  (2010)  [63] |  |  |  |  |  |  |  |  |  |  | Yes | Yes | Yes | Yes | Yes |  |  |  |  |  |  |  |  |  |  | 100 |
| Nair  (2011)  [62] |  |  |  |  |  |  |  |  |  |  | Yes | Yes | Yes | Can't tell | Yes |  |  |  |  |  |  |  |  |  |  | 80 |
| Nemeth  (2010)  [82] |  |  |  |  |  |  |  |  |  |  | Yes | Yes | Yes | Can't tell | Yes |  |  |  |  |  |  |  |  |  |  | 80 |
| Nobile  (2014)  [43] |  |  |  |  |  |  |  |  |  |  | Yes | Yes | Yes | Can't tell | Yes | Yes | Can't tell | Can't tell | Yes | Yes |  |  |  |  |  | 70 |
| O’Reilly  (2006)  [64] |  |  |  |  |  |  |  |  |  |  | Yes | Yes | Yes | No | Yes |  |  |  |  |  |  |  |  |  |  | 80 |
| Ozgun  (2010)  [65] |  |  |  |  |  |  |  |  |  |  | Yes | Yes | Yes | Can't tell | Yes |  |  |  |  |  |  |  |  |  |  | 80 |
| Parker  (2007)  [66] |  |  |  |  |  |  |  |  |  |  | Yes | Yes | Yes | Can't tell | Yes |  |  |  |  |  |  |  |  |  |  | 80 |
| Putnam  (2015)  [83] |  |  |  |  |  |  |  |  |  |  | Yes | Yes | Yes | Can't tell | Yes |  |  |  |  |  |  |  |  |  |  | 80 |
| Ribed  (2018)  [67] |  |  |  |  |  |  |  |  |  |  | Yes | Yes | No | Yes | Yes |  |  |  |  |  |  |  |  |  |  | 80 |
| Riggi  (2014)  [68] |  |  |  |  |  |  |  |  |  |  | Yes | Yes | Yes | Yes | Yes |  |  |  |  |  |  |  |  |  |  | 100 |
| Ritchie  (2004)  [69] |  |  |  |  |  |  |  |  |  |  | Yes | Yes | No | Yes | Yes |  |  |  |  |  |  |  |  |  |  | 80 |
| Rosenb  -erg  (2008)  [70] |  |  |  |  |  |  |  |  |  |  | Yes | Yes | No | Can't tell | No |  |  |  |  |  |  |  |  |  |  | 40 |
| Schwann  (2011)  [71] |  |  |  |  |  |  |  |  |  |  | Yes | Yes | Yes | No | Yes |  |  |  |  |  |  |  |  |  |  | 80 |
| Shapiro  (2018)  [72] |  |  |  |  |  |  |  |  |  |  | Yes | Yes | Can't tell | Can't tell | Yes |  |  |  |  |  |  |  |  |  |  | 60 |
| Suther  -land  (2014)  [73] |  |  |  |  |  |  |  |  |  |  | Yes | Yes | Yes | Can't tell | Yes |  |  |  |  |  |  |  |  |  |  | 80 |
| Tan  (2006)  [44] | Yes | Yes | Yes | Yes | Yes |  |  |  |  |  |  |  |  |  |  |  |  |  |  |  |  |  |  |  |  | 100 |
| Telfah  (2015)  [74] |  |  |  |  |  |  |  |  |  |  | Yes | Yes | Yes | Can't tell | Yes |  |  |  |  |  |  |  |  |  |  | 80 |
| Wax  (2007)  [75] |  |  |  |  |  |  |  |  |  |  | Yes | Yes | Yes | No | Yes |  |  |  |  |  |  |  |  |  |  | 80 |
| Whitman  (2008)  [76] |  |  |  |  |  |  |  |  |  |  | Yes | Yes | Yes | Yes | Yes |  |  |  |  |  |  |  |  |  |  | 100 |
| Willems  (2005)  [77] |  |  |  |  |  |  |  |  |  |  | Yes | Yes | Yes | Yes | Yes |  |  |  |  |  |  |  |  |  |  | 100 |
| Zanetti  (2003)  [78] |  |  |  |  |  | Yes | Can't tell | Yes | Yes | Yes |  |  |  |  |  |  |  |  |  |  |  |  |  |  |  | 80 |
| Zanotto  (2006)  [79] |  |  |  |  |  |  |  |  |  |  | Yes | Yes | Yes | Can't tell | Can't tell |  |  |  |  |  |  |  |  |  |  | 60 |
| Zhou  (2016)  [80] |  |  |  |  |  |  |  |  |  |  | Yes | Yes | Yes | Yes | Yes |  |  |  |  |  |  |  |  |  |  | 100 |
